# Can ACE-I Be a Silent Killer While Normal Renal Functions Falsely Secure Us?

**DOI:** 10.1155/2018/1852016

**Published:** 2018-07-09

**Authors:** Ahmed Abdelaal Ahmed Mahmoud, Mark Campbell, Margarita Blajeva

**Affiliations:** Department of Anaesthesia and Intensive Care Medicine, Tallaght University Hospital (Adelaide and Meath Incorporating National Children's Hospital), Ireland

## Abstract

The current case report represents a warning against serious hyperkalaemia and acidosis induced by ACE-I during surgical stress while normal renal function could deceive the attending anaesthetist. Arterial gas analysis for follow-up of haemoglobin loss accidentally discovered hyperkalaemia and acidosis. Glucose-insulin and furosemide successfully corrected hyperkalaemia after 25 minutes and acidosis after 3 hours. These complications could be explained by a deficient steroid stress response to surgery secondary to suppression by ACE-I. Event analysis and database search found that ACE-I induced aldosterone deficiency aggravated by surgical stress response with an inadequate increase in aldosterone secretion due to angiotensin II deficiency as a sequel of ACE-I leading to defective secretion of H+ and K+. Furosemide is recommended to secrete H+ and K+ compensating for aldosterone deficiency in addition to other antihyperkalaemia measures. Anaesthetising an ACE-I treated patient requires considering ACE-I as a potential cause of hyperkalaemia and acidosis.

## 1. Introduction

We present a case of accidentally discovered, unexpected intraoperative acidosis and hyperkalaemia in an ACE-I treated patient with no renal impairment or diabetes. Written consent was obtained from the patient for publication.

It is not uncommon for the anaesthetists to manage a hypertensive patient treated with Angiotensin Converting Enzyme Inhibitors (ACE-I) scheduled for either elective or emergency surgery. ACE-I are well known to be associated with a potential risk of perioperative side effects and complications as intraoperative hypotension and need for vasopressors, preoperative hyperkalaemia, perioperative drug-induced angioedema, and possible postoperative hypertension [1].

The risk of hyperkalaemia in ACE-I treated patients is higher in patients with type II diabetes or renal insufficiency. At least 10% of patients treated with ACE-I will experience even mild hyperkalaemia during their course of treatment with ACE-I [2]. The mechanism of ACE-I associated hyperkalaemia is mainly due to aldosterone deficiency with resultant decreased effect of aldosterone on collecting tubules leading to reduced excretion of potassium and decreased sodium reabsorption [2, 3].

## 2. Case Description

A 59-year-old male was scheduled for elective open retropubic prostatectomy for a benign enlarged prostate weighing approximately 65 grams. The patient's weight was 89 kg, ASA physical status II, diagnosed with essential hypertension two years ago, and controlled with ACE-I, Ramipril 10 mg once daily. No other morbidities were associated and no other medications were taken by the patient. The preoperative assessment did not reveal any other abnormality related to anaesthesia with normal vital signs, omitting Ramipril for 48 hours before the operation and normal baseline laboratory results including renal profile (creatinine 87 micromole/L, urea 7.9 mmol/L, Na 140 mmol/L, and K 4.1 mmol/L).

Following discussion with the patient and the surgical team, the anaesthetic plan was general anesthesia (GA) with postoperative patient-controlled analgesia (PCA) with morphine. Relatively uneventful induction of GA by propofol (2mg/kg), fentanyl (100 micrograms), and rocuronium (0.6 mg/kg) with endotracheal intubation, radial arterial cannulation for IBP monitoring, and two wide-bore peripheral cannulas (18G) were inserted. Induction was accompanied by hypotension (BP dropped from 112/68 to 73/46) and bradycardia (HR dropped from 78/min. to 38/min.) that required two successive doses of ephedrine each 6 mg were followed by restoration of BP and HR. Baseline arterial blood gas (ABG) after positioning was normal (Table 1). At 2 hours after the start of surgery, the estimated blood was about 350 ml and the urinary output (UOP) was 120 ml (over 2 hours) with mean arterial pressure (MAP) being maintained above 70 mmHg without further vasopressors required other than the initial 12 mg of ephedrine required immediately after induction. An arterial blood gas (2 hours after start of surgery) was initially performed for monitoring haemoglobin level showed hyperkalaemia (6.1 mmol/L) with acidosis (pH 7.33 and PCo2 6.2 kPa). The initial explanation was respiratory acidosis, and ventilation parameters were increased. Twenty-five minutes later ABG showed a decrease of PCo2 to normal with normal anion gap acidosis and increasing potassium to 6.5 mmol/L. Hyperkalaemia was treated with glucose-insulin (10 units of insulin added to 1 litre of glucose 10%) and mild hyperventilation and furosemide (20 mg bolus) with a change of the maintenance fluid from compound lactate solution to normal saline with the same rate (225ml/h). Forty minutes later, these measures had reduced potassium from 6.5 mmol/L to 4.1 mmol/L.

Despite normalization of potassium level (k 4.1 mmol/L) following the measures mentioned above, the acidosis persisted with maintained normal bicarbonate level and normal PCo2 (Table 1). From the time of normalized potassium, the acidosis required three hours to normalize which was two hours after recovery from GA. The presence of acidosis did not affect emergence from anaesthesia or recovery of the patient.

Postoperative follow-up of the renal function tests and electrolytes (Table 2) revealed normalization over a period of two days postoperatively with the patient restored intake of ACE-I on day one postoperatively with no effect on potassium level.

At the end of surgery, the estimated blood loss was about 635 ml, UOP was 700 ml (over an operative time of 4 hours), and the infused fluids included 450 ml of Hartman's solution (over the first 2 hours), 950 ml of 0.9% normal saline, and 500 ml of Gelofusine 4% (over the second 2 hours) in addition to 1 litre of glucose 10% with insulin. No blood transfusion was required and no MAP <70 mmHg was recorded.

## 3. Discussion

Systematic analysis of the event did not reveal any cause for the hyperkalaemia and acidosis except the history of treatment with ACE-I that may be associated with renal tubular acidosis (RTA), normal anion gap acidosis, and hyperkalaemia [2–13]; this explanation was supported by results from database search [1–15] including the effect of furosemide in hyperkalaemic RTA [11].

The patient did not receive blood transfusion and did not experience hypotensive events apart from a single induction related hypotension event that was corrected by a small dose of ephedrine; this excludes the possibility of either transfusion related or acute kidney injury due to hypotension as possible causes of hyperkalaemia. A single dose of propofol was used for induction of anaesthesia making the possibility of propofol infusion syndrome extremely unlikely.

The steroid hormone aldosterone acts on a mineralocorticoid receptor in the renal collecting ducts through a protein kinase mechanism leading to stimulation of Na+ reabsorption and active secretion of both H+ and K with potential risk for hyperkalaemia and acidosis in case of aldosterone deficiency [4, 5].

Angiotensin II is essential for the secretion of aldosterone [5] with ACE-I can produce a variable degree of hyporeninemic hypoaldosteronism or type IV renal tubular acidosis [6–10] characterized by metabolic acidosis and hyperkalaemia due to aldosterone deficiency.

Furosemide can be beneficial in type IV renal tubular acidosis by stimulation of K+ and H+ secretion from distal collecting tubules in case of hypoaldosteronism [11].

Typically, surgical induced stress response is associated with increased secretion of cortisol and aldosterone through stimulation of the hypothalamic-pituitary-adrenocortical axis (HPA-axis) with subsequent electrolyte changes including salt and water retention with hypokalaemia [12].

**Figure 1:**
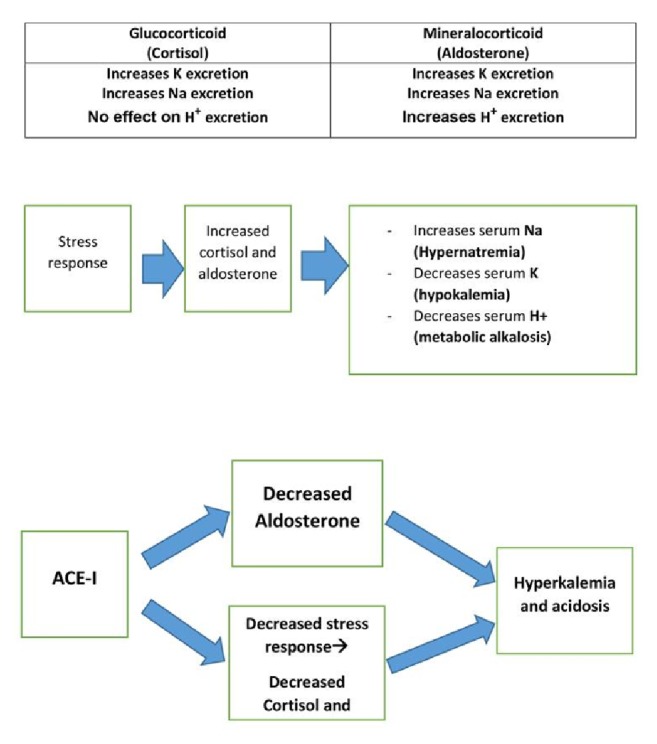
Pathophysiology of ACE-I induced hyperkalaemia and acidosis. ACE-I: Angiotensin Converting Enzyme Inhibitor.

Experimental research demonstrated that angiotensin II and ACE could intervene with the pituitary hormones secretion like corticotropin (ACTH) and enhances the stimulatory effects of corticotropin-releasing hormone (CRH), thus contributing to the stress-related activation of the hypothalamic-pituitary-adrenocortical (HPA) axis [13, 14]. Subsequently the inhibition of ACE with ACE-I will be associated decreased stress response, and with reduced cortisol secretion, this can aggravate the risk of hyperkalaemia and acidosis in ACE-I treated patients when exposed to surgical stress response (**Figure 1**).

Although, our patient stopped ACE-I 48 hours before surgery, it has been proved that aldosterone level does not change up to 15 days after cessation of Ramipril [16]. This augments the explanation that the hyperkalaemia can be due to the residual effect of ACE-I on aldosterone level.

It is known that renal impairment and diabetes can be associated with increased risk of hyperkalaemia and acidosis in ACE-I treated patients who may warn the anaesthetists to suspect, monitor, and detect such complication; however, we reported a case of unexpected hyperkalaemia and acidosis in a surgical patient treated with ACE-I with normal baseline renal function, with adequate response to dextrose insulin and furosemide and correction of both hyperkalaemia and acidosis. This event in our case report may be triggered by surgical stress response with deficient steroid secretion especially aldosterone; this is supported by the ability of furosemide to reverse the effects of aldosterone deficiency by secreting hydrogen ion and potassium.

Although the lactate level increased during the surgery, this cannot be explained by increased production as no haemodynamic instability was recorded but may be related to impaired excretion of lactate as a part of renal tubular acidosis.

The current patient experienced a mild increase in creatinine level and a mild drop in in glomerular filtration rate. The derangement in renal function postoperatively may be due to the use of furosemide intraoperatively, which has been recorded before to produce drop GFR [17].

Acidosis persisted despite potassium normalization with insulin, until successful correction was achieved with the diuretic effect of furosemide. Insulin administration produced correction of potassium but not acidosis as insulin may participate to acidosis [18] and this supports the fact that acidosis in our case was most probably due to the fact that renal tubular acidosis was induced by the preoperative use of ACE-I.

The possibility that catecholamines may shifted potassium intracellularly is not possible as catecholamines are also suppressed with ACE-I [19]

Patients on ACE-I are recommended for perioperative monitoring of potassium for early detection and management of such possible complications especially with unexplained ECG signs of hyperkalaemia and haemodynamic instability. In case of developing hyperkalaemia and acidosis, furosemide is recommended to secrete H+ and K+ compensating for aldosterone deficiency in addition to other antihyperkalaemia measures.

A limitation in our case report is that we did not measure the aldosterone level after the event.

## 4. Future Research

No previous studies [15] examined the effect of ACE-I on potassium and hydrogen ion during the surgical stress response; additionally, no information is available if ACE-I treated patients have a normal cortisol stress response or not in the perioperative period.

A future research study can assess the risk of hyperkalaemia and acidosis during the operative period in patients treated with ACE-I in addition to assessment of cortisol stress response in this category of patients.

## Figures and Tables

**Table 1:** Arterial blood gases following induction of anaesthesia and till discharge from the recovery unit.

**ABG**	**11:20**	**13:14**	**13:39**	**14:20**	**14:51**	**15:37**	**17:16**
**pH**	7.38	7.32	7.33	7.31	7.30	7.31	7.37
**PCO2 (kPa)**	5.5	6.2	6	5.9	6.1	6.2	5.5
**PO2 (kPa)**	12.5	20.8	22.3	26	20.8	15.5	11.7
**sO2 (%)**	95	97	98	98	98	98	95
**Base Excess(mmol/L)**	0	-2	-2	-4	-4	-3	-1
**Standard Bicarbonate (mmol/L)**	24	23	23	21	21	22	23
**Actual Bicarbonate (mmo/L)**	25	24	24	22	22	23	24
**Total haemoglobin (g/dl)**	12.9	13	12.9	11	9.8	10.4	10.5
**Carboxyhaemoglobin (%)**	0.2	0.1	0.2	0.5	0.6	1.2	0.7
**Whole blood glucose (mmol/L)**	5.9	7.1	8	16.9	9.2	7.2	8.9
**Methaemoglobin (%)**	1.5	1.5	1.6	1.6	1.8	1.9	1.8
**Sodium (mmol/L)**	139	137	136	133	138	138	138
**Potassium (mmol/L)**	4.2	6.1	6.5	4.6	4.0	4.1	3.8
**Lactate (mmol/L)**	0.7	0.8	1.0	1.5	1.8	2.0	0.7

**Table 2:** Renal function tests of the patient.

**Renal function test**	**Preoperative**	**Day 1 postoperative**	**Day 2 postoperative**
**Sodium (mmol/L)**	140	141	140
**Potassium (mmol/L)**	4.1	4.4	3.9
**Creatinine (umol/L)**	87	99	85
**Urea (mmol/L)**	7.9	6.6	5.4
_**e**_ **G** **F** **R**	80	69	82

_e_GFR: estimated glomerular filtration rate, calculated by Cockcroft and Gault modified equation.
